# Effect of a Water, Sanitation, and Hygiene Program on Handwashing with a Cleansing Agent among Diarrhea Patients and Attendants in Healthcare Facilities in the Democratic Republic of the Congo: A Randomized Pilot of the PICHA7 Program

**DOI:** 10.3390/ijerph21060659

**Published:** 2024-05-22

**Authors:** Alain Mwishingo, Kelly Endres, Lucien Bisimwa, Presence Sanvura, Blessing Muderhwa Banywesize, Jean-Claude Bisimwa, Camille Williams, Jamie Perin, Raissa Boroto, Gisèle Nsimire, Feza Rugusha, Freddy Endeleya, Pacifique Kitumaini, Claude Lunyelunye, Jessy Timsifu, Brigitte Munyerenkana, Justin Bengehya, Ghislain Maheshe, Cirhuza Cikomola, Christine Marie George

**Affiliations:** 1 Center for Tropical Diseases & Global Health, Université Catholique de Bukavu, Bukavu B.P 265, Democratic Republic of the Congo; alainmwish@gmail.com (A.M.); blessingmuderhwa@gmail.com (B.M.B.); boroto.iranga@ucbukavu.ac.cd (R.B.); giselensimire13@gmail.com (G.N.); pacifiquekitumaini@gmail.com (P.K.); claudelunye1@hotmail.fr (C.L.); jessytumsifu04@gmail.com (J.T.); brigmunyebik@gmail.com (B.M.); cikomola.cirhuza@ucbukavu.ac.cd (C.C.); 2 Department of International Health, Johns Hopkins Bloomberg School of Public Health, Baltimore, MD 21205, USAcwill302@jhmi.edu (C.W.); jperin@jhu.edu (J.P.); 3 Bureau de l’Information Sanitaire, Surveillance Epidémiologique et Recherche Scientifique, Division Provinciale de la Santé Sud Kivu, Ministère de la Santé, Bukavu B.P 265, Democratic Republic of the Congo; justinbengehya@gmail.com; 4 Faculty of Medicine, Catholic University of Bukavu, Bukavu B.P 265, Democratic Republic of the Congo; maheshe.balemba@ucbukavu.ac.cd

**Keywords:** Democratic Republic of the Congo, diarrheal disease, handwashing, healthcare facilities, behavior change

## Abstract

Healthcare-acquired infections are a major problem in healthcare facility settings around the world. The Democratic Republic of the Congo (DRC) has over 2 million diarrhea patients hospitalized each year. These healthcare settings become high-risk environments for spreading diarrheal illnesses such as cholera. The objective of the Preventative Intervention for Cholera for 7 Days (PICHA7) program is to develop evidence-based water, sanitation, and hygiene (WASH) interventions to reduce cholera and other severe diarrheal diseases in the DRC. The study objective was to evaluate the effectiveness of PICHA7 program delivery in increasing handwashing with a cleansing agent at stool/vomit- and food-related events in a healthcare facility setting among diarrhea patients and patient attendants. A pilot of the PICHA7 program was conducted among 284 participants in 27 healthcare facilities from March 2020 to November 2021 in urban Bukavu in the South Kivu Province of the DRC. The standard arm received the standard message provided in the DRC to diarrhea patients on the use of oral rehydration solution and a basic WASH message at healthcare facility discharge. The PICHA7 arm received the PICHA7 WASH pictorial module delivered by a health promoter focused on handwashing with a cleansing agent at the bedside of the diarrhea patient in the healthcare facility and provision of a soapy water bottle (water and detergent powder). Within 24 h of intervention delivery, a three-hour structured observation of handwashing practices at stool/vomit- and food-related events (key events) was conducted in healthcare facilities of diarrhea patients and their attendants. Compared to the standard arm, there was significantly more handwashing with a cleansing agent at key events in the PICHA7 arm (40% vs. 15%) (odds ratio: 5.04; (95% confidence interval (CI): 2.01, 12.7)). These findings demonstrate that delivery of the PICHA7 WASH pictorial module and provision of a soapy water bottle to diarrhea patients and their attendants presents a promising approach to increase handwashing with a cleansing agent among this high-risk population in healthcare facilities in the eastern DRC.

## 1. Introduction

In the Democratic Republic of the Congo (DRC), 85 million diarrhea episodes are estimated annually, with 11% of under-5 deaths due to diarrheal disease [1,2]. Cholera is endemic in the eastern region of the DRC, where each year outbreaks occur regularly [3]. Estimates suggest that the DRC accounts for between 5 and 14% of cholera cases each year worldwide [4]. Diarrheal disease transmission occurs primarily through inadequate water, sanitation, and hygiene (WASH), including contaminated water and poor hand hygiene practices [5].

Risk factors for cholera are generally fecal oral transmission pathways, with limited studies on the impact of WASH interventions in reducing cholera [6,7,8,9]. Protective interventions can be as simple and effective as using a sari to filter household drinking water, which resulted in a significant reduction in cholera in a trial conducted in Bangladesh [6]. Despite this, the literature has shown evidence gaps in the understanding of how to best design WASH interventions for high-risk populations [9]. In addition, a review of the WASH intervention guidelines used in cholera prevention programs found limited agreement across guidelines [7]. The authors of the review recommended a focus on evidence-based, resource-efficient approaches to cholera prevention.

Healthcare facilities are a high-risk setting for disease transmission globally, particularly in sub-Saharan Africa [10,11,12]. Adequate water, sanitation, and hygiene provision is important in healthcare facilities to prevent healthcare-acquired infections. Disease transmission in healthcare facilities is a concern for healthcare workers, patients, and for caregivers that accompany patients in the healthcare facility [13]. In the DRC, healthcare facilities often face staff and medical supplies shortages [14,15]. The Global Baseline Report on WASH in Health Care Facilities published in 2019 estimated that, in the DRC, 29% of the healthcare facilities had improved and useable sanitation coverage and only 24% of healthcare waste was adequately disposed of [16]. Urban coverage of improved sanitation was higher than in rural settings, and this coverage was also higher for hospital settings compared to non-hospital settings [16]. In formative research for this study, diarrhea patients reported that they perceived cholera treatment centers (CTCs) and health facilities as high-risk environments for disease transmission because they were viewed as unclean places [17]. In the DRC, individuals with acute diarrhea seek care at local healthcare facilities, often accompanied by household members and other caregivers (Personal communication: Dr. Lucien Bisimwa). Due to staff shortages in healthcare facilities, the family members and other caregivers of patients suffering from diarrheal diseases help to care for the patient during treatment (Personal communication: Alain Mwishingo), putting them at risk for disease transmission.

Research shows that household members of diarrhea patients are at an elevated risk of contracting diarrheal disease compared to the general population during the 7 days after the patient is admitted to a healthcare facility (100 times higher for cholera) [18,19,20]. Therefore, it is critical for diarrhea patients and those accompanying them in the healthcare facility to understand the importance of practicing WASH behaviors, especially hand hygiene. Proper hand hygiene practice in healthcare facilities is central to preventing the spread of disease [21]. While evidence is limited, hand hygiene in healthcare facilities in low- and middle-income countries is low [10,22]. Most studies assessing hand hygiene in healthcare facilities report on healthcare workers, with few studies evaluating hand hygiene among patients or their accompanying caregivers in low- or middle-income settings [23,24,25,26,27,28,29]. Interventions delivered in a healthcare facility setting have the potential to greatly impact hand hygiene behaviors for the caregivers accompanying patients in the healthcare facility [30]. In a study conducted by our research group in Bangladesh, we found that a targeted evidence-based handwashing intervention delivered to diarrhea patients and their caregivers while in the healthcare facility increased handwashing with soap when compared to patients and caregivers that did not receive the intervention, demonstrating the potential impact that healthcare facility interventions can have on patient and caregiver WASH [23].

To address diarrheal disease transmission among household members of diarrhea patients, we developed the Preventative Intervention for Cholera for 7 Days (PICHA7) program. This program targets WASH behaviors during the 7-day high-risk period for diarrheal disease transmission among cholera and severe diarrhea patients and their household members in partnership with the DRC Ministry of Health [17]. The program was named PICHA7 as “picha” means “picture” in Swahili, which represents the pictorial WASH modules included in this program, and “7” indicates the 7-day high-risk period for household members of diarrhea patients for subsequent diarrhea. The program was developed through extensive formative research, including semi-structured interviews and focus groups, and was informed by the Integrated Behavioural Model for Water, Sanitation, and Hygiene (IBM-WASH) framework for health behavior change [31]. The formative research identified facilitators of and barriers to the promoted WASH behaviors to reduce diarrheal disease transmission and inform the design of the PICHA7 program to target these identified facilitators and barriers [17]. The objectives of this study are (1) to describe the handwashing behaviors of diarrhea patients and their accompanying attendants in a healthcare facility setting, and (2) to assess the effectiveness of the PICHA7 WASH program on hand hygiene while in a healthcare facility. The PICHA7 WASH program fills an important evidence gap by determining whether a targeted WASH program for diarrhea patient households can increase handwashing with soap behaviors in a sub-Saharan African context.

## 2. Methods

### 2.1. Study Site and Population

This study was conducted across 27 healthcare facilities in 3 urban health zones of Bukavu, South Kivu province, in the eastern DRC. All 27 health facilities participated in this study. Data collection occurred between March 2020 and November 2021 as a part of the PICHA7 program pilot. 

To select the healthcare facilities included in this study, we used the list of private and public healthcare facilities in the province provided by the Ministry of Health. We conducted two methods of epidemiological surveillance of diarrhea patients. First, members of the research team visited healthcare facilities each day to recruit diarrhea patients. Second, previously identified healthcare facility staff contacted the research team by phone when new eligible patients were admitted to the healthcare facility. Diarrhea patients of all etiologies were included in our study.

Hospitalized diarrhea patients and their attendants were enrolled into our randomized pilot using the following eligibility criteria. Patient attendants were defined as anyone who was present with the diarrhea patient in a health facility during a structured observation, which includes relatives and friends. The inclusion criteria for index patients were that the patient (1) was admitted to a healthcare facility for diarrhea, defined as 3 or more liquid or loose stools within the past 24 h [32], (2) had stayed in their household for at least 3 nights prior to hospitalization, (3) had no functioning tap in the household (mostly slum areas), (4) had at least one functioning mobile phone in the household, and (5) had a child under 5 years of age in the household who planned to reside in the household with the diarrhea patient during the next 3 months. We excluded any patient who came to the health facility as an outpatient, or any patient who had already exceeded 24 h of hospitalization (all age groups). All accompanying patient attendants (mainly household members) present during study activities were included in structured observations.

### 2.2. Study Design and Intervention

Using a randomization table prepared by the study biostatistician, index patients and their household members were randomized to the PICHA7 arm or standard arm by health facility and ward to reduce information contamination. A 4:1 ratio (4 standard arm to 1 intervention household) was used, given we only needed a small sample size for our pilot study and this was nested within a larger observational study of handwashing practices in a health facility setting). The standard arm participants received the standard message provided in the DRC by the DRC Ministry of Health to diarrhea patients on the use of oral rehydration solution (ORS). The ORS message from the government of the DRC states: “Mix a packet of ORS with water, such as coconut water or rice water, and give it to the person suffering from severe diarrhea. If the diarrhea persists, take the individual with diarrhea to the health facility. Give the patient normal food.” The PICHA7 arm received the standard message and the PICHA7 program, which was developed through 18 months of community-centered formative research. These findings are included in our recently published manuscript [17]. The healthcare facility component of PICHA7 was provided at the bedside of the diarrhea patient to the patient and their accompanying household members and included (1) the pictorial WASH module, delivered by a health promoter, related to cholera and diarrheal disease transmission and handwashing with a cleansing agent; and (2) the delivery of a diarrhea prevention kit (one bottle of soapy water (water and detergent powder) and 1.5 L of chlorine-treated drinking water). A demonstration was conducted on how to wash one’s hands with soap using a handwashing station and how to prepare chlorine-treated water using a water vessel with lid and tap. 

The PICHA7 pictorial module contained WASH lessons that focus on understanding and preventing diarrhea, handwashing with soap at key times, water treatment using chlorine, and how to construct handwashing and drinking water stations. In the health facility PICHA7 pictorial module, there was also a lesson explaining how to receive mobile calls and text messages from our WASH mobile health program after discharge.

### 2.3. Data Collection

In our study protocol, 3 h structured observation was conducted in healthcare facilities by a trained research officer approximately 24 h after PICHA7 or standard intervention delivery to observe handwashing behaviors among diarrhea patients and patient attendants at key events. Observation data were collected using a structured questionnaire form on a netbook computer according to our previously published methods [33,34]. Key events recorded during structured observation included (1) before food preparation (food), (2) before eating (food), (3) before feeding someone (food), (4) after going to the toilet (stool/vomit), (5) after disposing of stool (stool/vomit), (6) after vomiting (stool/vomit), (7) after cleaning vomit or stool, and (8) after washing the anus of children (stool/vomit). We recorded whether hand washing was completed with one or two hands and the type of cleansing agent used. The cleansing agents observed were bar soap, liquid soap, soapy water (water and detergent powder mixed together), chlorinated water, and hand sanitizer. Patient attendants were defined as individuals present with the patient in the healthcare facility at the time structured observation was conducted. All handwashing stations in the CTC at the Provincial Hospital of Bukavu were categorized as containing chlorinated water based on the standard operating procedure of the hospital evaluated in our previous study (Personal communication, Alain Mwishingo). Handwashing stations in other wards and at other healthcare facilities were considered chlorinated if chlorine-treated water signage was present at the handwashing station. Research officers could not be blinded to the study arm of study participants because the intervention had visible components (e.g., the soapy water bottle).

Healthcare facility spot checks were conducted by trained research officers after each structured observation in the healthcare facility ward where an index patient was hospitalized. Ward type and the number of functional beds, patients, handwashing stations, and functional handwashing stations were assessed. To be admitted to the pediatric ward of a healthcare facility, patients had to be under 18 years of age. Those 18 years of age or older were admitted to an internal medicine ward. Those admitted to the intensive care unit were patients with life-threatening conditions or at risk of acute complications requiring close monitoring. Functional beds were defined as beds that are useable, i.e., not including beds that are broken, covered in supplies, etc. Handwashing stations were considered non-functional if the tap was broken, no water was present, or no cleansing agent was present. 

### 2.4. Data Analysis

Our main study aim was to understand handwashing behaviors among diarrhea patients and patient attendants in a healthcare facility setting and the impact of the PICHA7 program intervention on handwashing. We defined handwashing with a cleansing agent as observed handwashing of both hands with a cleansing agent at food and stool/vomit key times at least once during the 3 h structured observation. At least one hand washed with a cleansing agent and at least one hand washed with only water (non-chlorinated) were also assessed. Descriptive statistics were used to examine participant characteristics and handwashing with a cleansing agent among participants over 2 years of age, overall and by study arm. We compared descriptive statistics using Pearson’s chi-square test, utilizing Fisher’s exact test when there were fewer than 5 observations in a category. Handwashing events were further stratified by patients and patient attendants and type of key event (food- or stool/vomit-related event). Handwashing with a cleansing agent in the PICHA7 and standard arms among participants over 2 years of age was compared using logistic regression with generalized estimating equations and an exchangeable correlation structure to account for clustering at the household level (patient and attendants). For logistic regression models, handwashing with a cleansing agent was the outcome and study arm was the predictor. This was compared for all participants (diarrhea patients and patient attendants) and patient attendants only. Finally, we examined the availability of functional handwashing stations and materials in healthcare facilities using descriptive statistics. Analyses were completed using SAS 9.4 software (Cary, NC, USA). 

### 2.5. Ethics

The National Health Ethics Committee (NHEC) in the DRC, the Catholic University of Bukavu, and the Johns Hopkins Bloomberg School of Public Health IRB approved all the study protocols. At recruitment, the informed consent document was read to and consent obtained from all study participants (diarrhea patients and household members of diarrhea patients). Parental permission was obtained for children under 17 years of age in addition to consent from individuals 12 to 17 years of age.

## 3. Results

A total of 284 participants were observed in this study, 39% (110/284) of whom were index diarrhea patients and 61% (174/284) of whom were patient attendants (Table 1). There were 67 participants in the PICHA7 arm and 217 participants in the standard arm. Sixty-nine percent of all the participants (197/284) were women. The average ages of the patient attendants in the standard and PICHA7 arms were 23.7 years (standard deviation (SD): 14.2) and 22.6 years (SD: 16), respectively.

A total of 113 spot checks were conducted across 27 healthcare facilities (Table 2). We conducted 46% (52/113) of the spot checks at the Provincial Referral Hospital of Bukavu. Forty-one percent (11/27) of the health facilities had more than one ward type observed. The pediatric wards had the highest number of enrolled diarrhea cases (38% (43/113)), followed by the CTC in the Provincial Referral Hospital of Bukavu (20% (23/113)), internal medicine wards (6% (7/113)), and intensive care wards (2% (2/113)). The average number of functional beds per ward was 8.2 (SD: 6.1, range: 1–25). We observed on average one functional handwashing station (SD: 0.79, range: 0–4) per ward. In total, 88% (115/130) of the handwashing stations were functional.

Observed handwashing with a cleansing agent was significantly higher in the PICHA7 arm than in the standard arm among all the participants (attendants and patients) (40% (16/40) and 15% (21/136), respectively) (Table 3), (odds ratio (OR): 5.04; 95% confidence interval (CI): 2.01, 12.7) (Table 4). At food events, handwashing with a cleansing agent was also significant for all the participants at 35% (13/37) in the PICHA7 arm compared to 12% (15/128) in the standard arm (OR: 6.57; 95% CI: 2.23, 19.3). At all stool/vomit-related events, handwashing with a cleansing agent was also significantly higher for all the participants at 37% (7/19) in the PICHA7 arm compared to 14% (9/63) in the standard arm (OR: 3.42; 95% CI: 1.10, 10.6). Additional information about individual handwashing events and handwashing stations used is available in Table S1. 

In the PICHA7 arm, 20% (28/139) of the participants used a soapy water bottle at key handwashing events compared to 1% of the standard arm participants (5/517) (Table 5). Chlorinated water was used by 1% (2/139) of the PICHA7 arm participants and 3% (13/517) of the standard arm participants at a key handwashing event. 

## 4. Discussion

In this study, we assessed handwashing behaviors among diarrhea patients and patient attendants and the impact of the PICHA7 program on handwashing behaviors in a healthcare facility setting in the eastern DRC. In the standard arm, overall handwashing with a cleansing agent was low (15%). Patients and attendants receiving the PICHA7 program had higher handwashing with a cleansing agent in a healthcare facility setting compared to those receiving the standard message given in the DRC on ORS. These results demonstrate the urgent need for targeted WASH interventions to increase handwashing with a cleansing agent at stool-, vomiting-, and food-related events for diarrhea patients and their household members in healthcare facility settings in our study setting in the DRC. These findings suggest that delivery of the PICHA7 WASH program may serve as a potentially promising approach to increase handwashing with a cleansing agent compared to the message given on ORS in this high-risk setting for diarrheal disease transmission among diarrhea patients and their attendants. 

Patients and attendants receiving the PICHA7 program performed more handwashing with a cleansing agent in a healthcare facility setting compared to standard message delivery at stool-, vomiting-, and food-related events. One potential reason for this finding is the theory-informed and evidence-based development of the PICHA7 intervention, which engaged community members to develop a targeted WASH program tailored to a high-risk population for diarrheal diseases [17]. The development of PICHA7 was grounded in health behavior theory and was responsive to the facilitators of and barriers to WASH behaviors among diarrhea patients and their household members. There are no randomized pilots or randomized controlled trials (RCTs) in an African low- and middle-income country (LMIC) setting that we are aware of that have investigated the association between WASH delivery in a health facility setting to diarrhea patients and observed handwashing with soap among patients and patient attendants. In Kenya, WASH was delivered in a health facility setting to caregivers of children and health workers together with HIV prevention programs [35]. This program, however, did not have a control arm and did not conduct structured observation to observe handwashing events. Studies in Uganda and Malawi provided a WASH package in a health facility setting to pregnant mothers as part of antenatal care and found significantly higher self-reporting of water treatment in the intervention compared to the comparison group and to baseline levels among intervention participants (observed handwashing outcomes were not assessed) [36,37]. In Bangladesh and Cambodia, studies have conducted structured observation of handwashing events in health facilities to inform intervention development and have observed very low rates of handwashing with a cleansing agent [38,39]. Future RCTs or randomized pilots similar to the PICHA7 pilot are needed in a sub-Saharan Africa LMIC setting.

Our findings are consistent with a study we conducted in Bangladesh regarding the Cholera-Based Intervention for 7 days (CHoBI7), where a similar targeted WASH intervention was delivered to diarrhea patients and their household members in a health facility setting [23]. In this study, conducted in urban slum areas of Dhaka, Bangladesh, there was significantly more handwashing with soap at food- and stool-related key events in the ChoBI7 arm, where a pictorial WASH module and a soapy water bottle were delivered by a health promoter compared to the standard arm (51% vs. 25%), similar to the present study. Consistent with our findings, the availability of materials for handwashing has been shown to increase handwashing behavior [40,41]. Delivery of materials such as the soapy water bottle in healthcare facility settings, when combined with a WASH communication program, can serve as a potentially promising approach to increase hand hygiene in this high-risk healthcare facility setting. 

The PICHA7 program targets the 7-day period after a diarrhea patient is admitted to a healthcare facility for treatment, when household members are at highest risk for subsequent diarrheal diseases [18,19]. Delivering the PICHA7 program in a healthcare facility setting allows us to target diarrhea patient households during the period of time when perceived susceptibility to diarrheal diseases is likely the highest and when the perceived benefits of WASH are likely high [42]. Consistent with our findings, a recent study in the DRC found that delivery of a targeted WASH program delivered in a health facility setting significantly decreased household drinking water contamination [43]. Furthermore, the findings from the ongoing RCT of the PICHA7 program (NCT05166850) will report the impact of continued WASH programming (including home visits and weekly mobile health messages) on diarrheal disease and sustained WASH outcomes in households for a 12-month period in the eastern DRC. 

Handwashing with a cleansing agent at stool-, vomit-, and food-related events among both patients and their attendants was very low prior to the delivery of the PICHA7 pilot intervention. Only 14% of the patients and 16% of the patient attendants were observed to wash their hands with a cleansing agent at least once during the structured observation period in the standard arm despite high availability of functional handwashing stations (88%). To our knowledge, this is the first report of patient and attendant handwashing in the DRC using structured observation. There is a high risk of diarrheal disease spread in healthcare facilities since attendants are coming into frequent contact with the feces or vomit of patients, highlighting the importance of targeted WASH interventions for patients and their attendants [13]. Availability of handwashing materials in DRC healthcare facilities serves as an important barrier to handwashing in this context [44,45] and was discussed by participants during formative research development for the PICHA7 program [17]. Given the history of the Ebola virus in the eastern DRC, many cholera treatment centers in the DRC provide chlorinated water for handwashing [17,46]. However, there are often challenges with ensuring the correct dosage of chlorine, and, in some cases, patients and household members may not be aware that the water provided for handwashing is treated with chlorine to cleanse hands, as was found at our study site in the DRC [17]. In resource-constrained settings such as the DRC, utilization of cholera outbreak prediction methods (such as those developed for climate and weather patterns) could be helpful to ensure provision of handwashing materials during high-risk outbreak periods [47,48].

This study had several strengths: first, the randomized design for study arm assignment, reducing bias associated with intervention assignment; second, the use of structured observations instead of the more commonly used self-reported measures, which are prone to reporting bias; and last, our focus on patients and attendants, building on previous studies that mostly focused on healthcare workers [10,22]. This study also had limitations. First, we did not assess hand contamination, which could have served as a valuable measure to assess actual fecal contamination on hands instead of solely relying on structured observation. Second, we did not assess the chlorine concentration of each handwashing station present in the healthcare facility wards and instead used signage or ward type as a proxy. Third, although structured observation is preferable over self-reported handwashing measures, this method may still contain bias due to the Hawthorne effect [49]. In addition, research officers conducting structured observation could not be blinded to the study arm of the participants because of the visible components of the intervention (e.g., the soapy water bottle), and, beyond research staff training and refresher training, we did not use a method of standardization like videotaping for structured observation. Finally, our small sample size limited our ability to stratify our study findings by type of health facility (e.g., private vs. public) and participant type.

## 5. Conclusions

Patient and patient attendants receiving the PICHA7 WASH program performed significantly more handwashing with a cleansing agent among diarrhea patients and attendants in a health facility setting compared to those receiving the standard message on ORS in the eastern DRC. We are currently partnering with the Ministry of Health in the DRC to develop a scaling plan for the PICHA7 program in the country. Our findings suggest that the targeted PICHA7 program presents a promising approach to increase handwashing with a cleansing agent in a high-risk setting for the spread of diarrheal diseases in the DRC. 

## Figures and Tables

**Table 1:** Characteristics of diarrhea patients and accompanying patient attendants in health facilities in Bukavu, DRC, in the standard and the PICHA7 intervention arms.

	Overall (*N* = 284)		Standard Arm (*N* = 217)		PICHA7 Arm (*N* = 67)	
	%	*N*	%	*N*	%	*N*
Diarrhea patients	39%	110	40%	87	34%	23
Patient attendants	61%	174	60%	130	66%	44
Female	69%	197	69%	149	72%	48
Age (years, all)		247		190		57
Mean ± SD (min–max)	17.5 ± 16.9 (0–65)		17.5 ± 16.7 (0–65)		17.3 ± 17.6 (0–60)	
0–2	29%	71	27%	52	33%	19
2–5	10%	25	12%	22	5%	3
5–12	6%	16	5%	10	11%	6
>12	55%	135	56%	106	51%	29
Age (years, patients)		101		80		21
Mean ± SD (min–max)	8.85 ± 16.2 (0–63)		9.04 ± 16.2 (0–63)		8.14 ± 16.9 (0–58)	
0–2	53%	54	49%	39	71%	15
2–5	19%	19	21%	17	10%	2
5–12	7%	7	9%	7	0%	0
>12	21%	21	21%	17	19%	4
Age (years, patient attendants)		146		110		36
Mean ± SD (min–max)	23.4 ± 14.6 (0–65)		23.7 ± 14.2 (0–65)		22.6 ± 16.0 (0–60)	
0–2	12%	17	12%	13	11%	4
2–5	4%	6	5%	5	3%	1
5–12	6%	9	3%	3	17%	6
>12	78%	114	80%	89	69%	25
Ward Type		256		197		59
Cholera treatment center	20%	53	24%	47	10%	6
Internal medicine	4%	10	4%	8	3%	2
Pediatrics	40%	101	34%	68	56%	33
Intensive care	2%	4	2%	4	0%	0
Other	34%	88	36%	70	31%	18

SD = standard deviation. Patient attendants are defined as anyone who was present with the diarrhea patient in a health facility during a structured observation, which includes friends and relatives.

**Table 2:** Characteristics of health facility wards observed with diarrhea patients in Bukavu, DRC.

	%	Mean ± SD (min–max)	*n*	*N*
Spot checks conducted				113
Health facilities observed				27
Ward type				
Cholera treatment center	20%		23	
Internal medicine	6%		7	
Pediatrics	38%		43	
Intensive Care	2%		2	
Other	34%		38	
Functional beds		8.2 ± 6.1 (1–25)		926
Patients in ward		2.0 ± 2.3 (1–16)		225
Handwashing stations		1.2 ± 0.86 (0–4)		130
Functioning handwashing stations		1.0 ± 0.79 (0–4)		115

SD = standard deviation; functional beds were defined as beds that are useable, i.e., not including beds that are broken, covered in supplies, etc.; handwashing stations were considered non-functional if the tap was broken, no water was present, no cleansing agent was present, etc.; *n* indicates the total in a specific category across all spot checks; *N* indicates the total across all spot checks; mean provided per individual spot check.

**Table 3:** Handwashing among patients and patient attendants over 2 years of age at health facilities in Bukavu, DRC, in the standard and the PICHA7 intervention study arms.

	Standard Arm				PICHA7 Arm			
	% (*n*)			*N*	% (*n*)			*N*
Participants Handwashing with a Cleansing Agent	1	2	3		1	2	3	
All key events								
All participants	15% (21)	19% (26)	13% (17)	136	40% (16)	45% (18)	15% (6)	40
Patients	14% (5)	17% (6)	3% (1)	36	40% (2)	60% (3)	0% (16)	5
Patient attendants	16% (16)	20% (20)	16% (16)	100	40% (14)	43% (15)	17% (6)	35
All food events								
All participants	12% (15)	16% (20)	12% (15)	128	35% (13)	41% (15)	16% (6)	37
Patients	13% (4)	16% (5)	0% (0)	32	40% (2)	60% (3)	0% (0)	5
Patient attendants	11% (11)	17% (15)	17% (15)	96	34% (11)	38% (12)	19% (6)	32
All stool/vomit events								
All participants	14% (9)	14% (9)	6% (4)	63	37% (7)	37% (7)	0% (0)	19
Patients	12% (2)	12% (2)	6% (1)	17	0% (0)	0% (0)	0% (0)	2
Patient attendants	15% (7)	15% (7)	7% (3)	46	41% (7)	41% (7)	0% (0)	17

1 = washing both hands with a cleansing agent; 2 = at least one hand washed with a cleansing agent; 3 = at least one hand washed with only water; *N* indicates total number of participants; cleansing agents include bar soap, liquid soap, soapy water, chlorinated water, and hand sanitizer; patient attendants are defined as anyone who was present with the diarrhea patient in a health facility during a structured observation, which includes friends and relatives; food-related events include (1) before food preparation, (2) before eating, (3) before feeding someone, and 4) before breastfeeding; stool/vomit-related events include (1) after going to the toilet, (2) after eliminating stool, (3) after vomiting, and (4) after washing the anus of children.

**Table 4:** Logistic regression analysis comparing handwashing with a cleansing agent in the PICHA7 intervention arm to the standard arm during structured observation at health facilities in Bukavu, DRC.

Participants Washing Both Hands with a Cleansing Agent	*N*	*n*	OR (95% CI)
All events			
All participants	176	37	5.04 (2.01, 12.7)
Patient attendants	135	30	5.27 (2.01, 13.8)
Food events			
All participants	165	28	6.57 (2.23, 19.3)
Patient attendants	128	22	6.27 (2.06, 19.1)
Stool/vomit events			
All participants	82	16	3.42 (1.10, 10.6)
Patient attendants	63	14	3.68 (1.02, 13.3)

OR = odds ratio; CI = confidence interval; all events includes both stool/vomit and food events; *N* = number of participants with key event; *n* = number of key events with correct handwashing (both hands washed with cleansing agent; cleansing agents include bar soap, liquid soap, soapy water, chlorinated water, and hand sanitizer); patient attendants are defined as anyone who was present with the diarrhea patient in a health facility during a structured observation, which includes friends and relatives; food-related events include (1) before food preparation, (2) before eating, (3) before feeding someone, and (4) before breastfeeding; stool/vomit-related events include (1) after going to the toilet, (2) after eliminating stool, (3) after vomiting, (4) after cleaning vomit or stool, and (5) after washing the anus of children.

**Table 5:** Cleansing agents used during handwashing events among all participants over 2 years of age at health facilities in Bukavu, DRC, in the standard and the PICHA7 intervention study arms.

	Standard Arm (*N* = 517)		PICHA7 Arm (*N* = 139)	
	*n*	%	*n*	%
Water (non-chlorinated)	35	7%	40	29%
Soapy water bottle	0	0%	22	16%
Soapy water bottle (provided by UCB)	5	1%	6	4%
Soap (bar)	10	2%	2	1%
Soap (liquid)	4	1%	0	0%
Soap (detergent powder)	0	0%	1	1%
Hand sanitizer	4	1%	0	0%
Water (chlorinated)	13	3%	2	1%
Other	1	0%	0	0%

*N* indicates the total number of participants with key events during structured observation; *n* indicates the number of participants that used the cleansing agent for handwashing; more than one handwashing agent can be used per participant.

## Data Availability

Anonymized data may be made available upon request. Requests should be directed to Christine Marie George (cgeorg19@jhu.edu).

## Supplementary Materials

The following supporting information can be downloaded at https://www.mdpi.com/article/10.3390/ijerph21060659/s1, Table S1: Characteristics of diarrhea patients’ and accompanying patient attendants’ key handwashing events in the standard arm compared to the PICHA7 intervention arm during structured observation at health facilities in Bukavu, DRC.
