# Supplementary material for: Effect of a Water, Sanitation, and Hygiene Program on Handwashing with a Cleansing Agent among Diarrhea Patients and Attendants in Healthcare Facilities in the Democratic Republic of the Congo: A Randomized Pilot of the PICHA7 Program
Source: Int J Environ Res Public Health. 2024 May 22;21(6):659. doi: 10.3390/ijerph21060659 (PMC11204100; doi:10.3390/ijerph21060659)
Supplement: Supplementary file 1 [file ijerph-21-00659-s001.zip › ijerph-2891184-supplementary.pdf]

Supplemental Table S1. Characteristics of diarrhea patients and accompanying patient attendants handwashing events in the standard to the PICHA7 intervention arm during structured observation at health facilities in Bukavu, DRC

	**Overall**		**Standard Arm**		**PICHA7 Arm**	
	%	n	%	n	%	n
Handwashing events		656		517		139
Patients	19%	126	20%	104	16%	22
Patient attendants	81%	530	80%	413	85%	117
Event type						
Food event	79%	515	78%	404	80%	111
Stool/vomit event	21%	141	22%	113	20%	28
Hands washed	18%	119	14%	71	35%	48
Handwashing with cleansing agent	9%	59	5%	28	22%	31
Handwashing station type						
Faucet/tap and basin	2%	10	2%	9	1%	1
Plastic bucket and soap	2%	10	1%	6	3%	4
Plastic cup and bowl	3%	17	2%	8	6%	9
Other	7%	43	6%	30	9%	13

SD = standard deviation.

Participants may have multiple handwashing events per structured observation.

Patient attendants are defined as anyone who was present with the diarrhea patient in a health facility during a structured observation, which includes friends and relatives.

All percentages out of total handwashing events.
